# Unobtrusive Behavioral and Activity-Related Multimodal Biometrics: The ACTIBIO Authentication Concept

**DOI:** 10.1100/tsw.2011.51

**Published:** 2011-03-01

**Authors:** A. Drosou, D. Ioannidis, K. Moustakas, D. Tzovaras

**Affiliations:** ^1^ Department of Electrical Engineering, Imperial College London, London, UK; ^2^ Informatics and Telematics Institute, Centre for Research and Technology Hellas, Thermi-Thessaloniki, Greece

**Keywords:** biometrics, behavioral biometrics, physiological biometrics, image analysis, activity-related recognition, event detection, activity recognition, clustering, HMM, classification, authentication

## Abstract

Unobtrusive Authentication Using ACTIvity-Related and Soft BIOmetrics (ACTIBIO) is an EU Specific Targeted Research Project (STREP) where new types of biometrics are combined with state-of-the-art unobtrusive technologies in order to enhance security in a wide spectrum of applications. The project aims to develop a modular, robust, multimodal biometrics security authentication and monitoring system, which uses a biodynamic physiological profile, unique for each individual, and advancements of the state of the art in unobtrusive behavioral and other biometrics, such as face, gait recognition, and seat-based anthropometrics. Several shortcomings of existing biometric recognition systems are addressed within this project, which have helped in improving existing sensors, in developing new algorithms, and in designing applications, towards creating new, unobtrusive, biometric authentication procedures in security-sensitive, Ambient Intelligence environments. This paper presents the concept of the ACTIBIO project and describes its unobtrusive authentication demonstrator in a real scenario by focusing on the vision-based biometric recognition modalities.

### INTRODUCTION

The use of biometrics for access control in restricted infrastructures has been extensively researched during the last 4 decades. Biometrics measures the unique physical or behavioral characteristics of individuals as a means to recognize or authenticate their identity. Common physical biometrics include fingerprints; hand or palm geometry; and retina, iris, or facial characteristics. Behavioral characteristics include signature, voice (which also has a physical component), keystroke pattern, and gait. Although some technologies have gained more acceptance than others, it is beyond doubt that the field of access control and biometrics as a whole show great potential for use in end-user segments and for covering areas such as airports, stadiums, and defense installations, but also industry and corporate workplaces where security and privacy are required.

However, current biometric security systems exhibit various shortcomings despite their wide acceptance in commercial applications[1,2]. One such shortcoming is the *de facto* exclusion or discrimination of people by the system whose biometrics cannot be recorded well for the creation of the database reference (e.g., people whose fingerprints do not print well or people who miss the required limb or feature). In that respect, the research on new biometrics that use features that exist in every human, thus rendering them applicable to the greatest possible percentage of the population, becomes very important.

Late technologies in biometrics resemble more natural ways of recognizing people. For instance, friends do not recognize each other by examining each others' palms or fingerprints. Thus, similar to the ways or techniques that humans use to recognize each other, modern trends in biometrics indicate the recognition of dynamic face grimaces, gait, movements, etc. In other words, they tend to recognize "aliveness" rather than static features such as fingerprint traits or irises. Following this principle, behavioral biometrics is related to specific actions and the way that each person executes them. In this respect, spoofing attacks to biometric systems that usually use artificially made features (fingerprints, photographs, etc.)[3] will fail. Due to the nature of dynamic indicators that describe a person's internal physiology and external behavior, an authentication system that uses them performs *de facto* and reliably an aliveness check of that person.

Moreover, identity fraud is possible when authentication takes place instantaneously and only once. An attacker can bypass the biometric authentication system and continue undisturbed. Given this, a cracked or stolen biometric system presents a difficult problem. Unlike passwords or smart cards that can be changed or reissued, absent serious medical intervention, a fingerprint or iris is forever. Once an attacker has successfully forged those characteristics, the end user must be excluded from the system entirely, raising the possibility of enormous security risks and/or reimplementation costs. Static physical characteristics can be digitally duplicated; for example, the face could be copied using a photograph, a voice print using a voice recording, and the fingerprint using various forging methods. In addition, static biometrics could be intolerant of changes in physiology. For instance, the voice could be altered by a cold, while face recognition systems are susceptible to changes under ambient light conditions or the pose of the subject. Behavioral and physiological dynamic indicators as a response to specific stimuli could address these issues and enhance the reliability and robustness of biometric authentication systems when used in conjunction with the usual biometric techniques. Additionally, continuous and repeated authentication of a person (in the controlled environment) is possible because of the nature of dynamic traits.

Furthermore, given that most of the existing biometric systems are unimodal systems, it makes them more vulnerable to theft attempts since an attacker can easily gain access by stealing or bypassing a single biometric feature. In the same concept, unimodal biometric systems have to contend with a variety of problems, such as noisy data, intraclass variations, restricted degrees of freedom, nonuniversality, spoof attacks, and unacceptable error rates, i.e., it is estimated that approximately 3% of the population does not have legible fingerprints[4]. This kind of biometric system may not always meet performance requirements, may exclude large numbers of people, and may be vulnerable to everyday changes and lesions of the biometric feature. Because of this, the development of systems that integrate two or more biometrics is emerging as a trend.

On the other hand, some of the aforementioned limitations can be addressed by deploying multimodal biometric systems that integrate the evidence presented by multiple sources of information. A multimodal biometric system uses multiple applications to capture different types of biometrics. This allows the integration of two or more types of biometric recognition and verification systems in order to meet stringent performance requirements. A multimodal system can combine any number of independent biometrics and overcome some of the limitations arising when using just one biometric as the verification tool. Multiple biometrics could consist of different types of biometrics, such as combining facial and iris recognition. Particularly, the use of behavior, physiology, and signals from either wearable sensors or sensors integrated into the infrastructure in a multimodal setup might be of special interest considering that they share the most part of the signal acquisition system. Experimental results have demonstrated that the identities established by systems that use more than one biometric[5] could be more reliable, be applicable to large target populations, and improve response time.

Last but not least, a major shortcoming of all biometrics is the obtrusive process of obtaining the biometric feature. The subject has to stop, go through a specific measurement procedure (which, depending on the biometric, can be very obtrusive), wait for a period of time, and then get clearance after authentication is positive. Emerging biometrics such as gait recognition[3], dynamic body motion recognition, and technologies such as automated face/gestures dynamics detection, as well as biometrics measured by sensors either worn by the user or transparently integrated into the infrastructure, can potentially allow the nonstop (on-the-move) authentication or even identification that is unobtrusive and transparent to the subject and become part of an Ambient Intelligence (AmI) environment. In this respect, the identity of the users will be initially established based on their behavioral biometric features and it will be continuously validated based on activity-related signals that can be captured in an unobtrusive way.

### ACTIBIO CONCEPT

The ACTIBIO (ACTIvity-Related and Soft BIOmetrics) project has researched and developed a completely new concept in biometric authentication, i.e., the extraction of biometric signatures based on the response of the user to specific stimuli, while performing specific work-related activities, such as answering the phone, talking into the microphone, controlling an office panel, etc. Each activity is stimulated by environmental events, i.e., ringing of the phone or the announcement of an alarm message through the microphone.

The novelty of the approach lies in the fact that the measurements from several biometric subsystems (i.e., modalities) that are used for authentication correspond to the response of the person to specific events being, however, fully unobtrusive and also fully integrated in an AmI infrastructure. Thus, the current system implements a multimodal approach, fusing information from various sensors capturing either the dynamic behavioral profile of the user (face, gesture, gait, body dynamics) or the physiological response of the user to events. Within the ACTIBIO project, novel activity-related and soft biometric technologies for improving security, trust, and dependability of “always-on” networks and service infrastructures have been developed. (The term “always on” refers to any system [PC, infrastructure device, etc.] that is online and ready to go 24 h a day. Nothing has to be turned on or dialed up in order to start it.)

The biometric modalities that are initially considered compatible with the user’s behavioral analysis in the project are the face, the gait, the body poses, as well as some “soft” biometrics (e.g., height), and special biometrics that can be collected via the use of unobtrusive sensors, i.e., a sensing seat[6] that is able to extract anthropometric profiles based on the user’s weight distribution on the seat and the seat’s cover deformation. Additionally, activity-related biometrics (i.e., prehension movements) are studied in conjunction with physiological biometrics extracted from electroencephalography (EEG) and electrocardiography (ECG) using wireless sensors.

In general, the novelty lies in the introduction of invariant physiological activity-related biometrics that are combined with unobtrusive behavioral and soft biometrics by using a triplet of sensors: (a) an improved sensing seat, (b) a wireless physiological sensor, and (c) stereo cameras.

In this respect, the monitoring of actions performed by the user is triggered by a dedicated event detector, occasionally augmented by wired usb-sensors. The user authentication is based on signals with dynamic nature (focusing on the behavioral part), which significantly vary among different users Further, increased safety levels are boosted via the continuous, on-the-move behavioral and physiological analysis of the data provided by several modalities. In the same concept, multimodal signal analysis techniques are used for the detection, while user-specific activity patterns are processed from a system fully unobtrusive and fully integrated in AmI environments.

### ARCHITECTURE

An open, modular, and efficient system architecture has been designed in order to address the different applications and systems of the ACTIBIO project. The overall framework focuses on the definition of nodes (independent modalities) in the network, their functionalities, the definition of internode communication protocols, and the information flow between nodes. A proposed architecture is presented in Fig. 1.

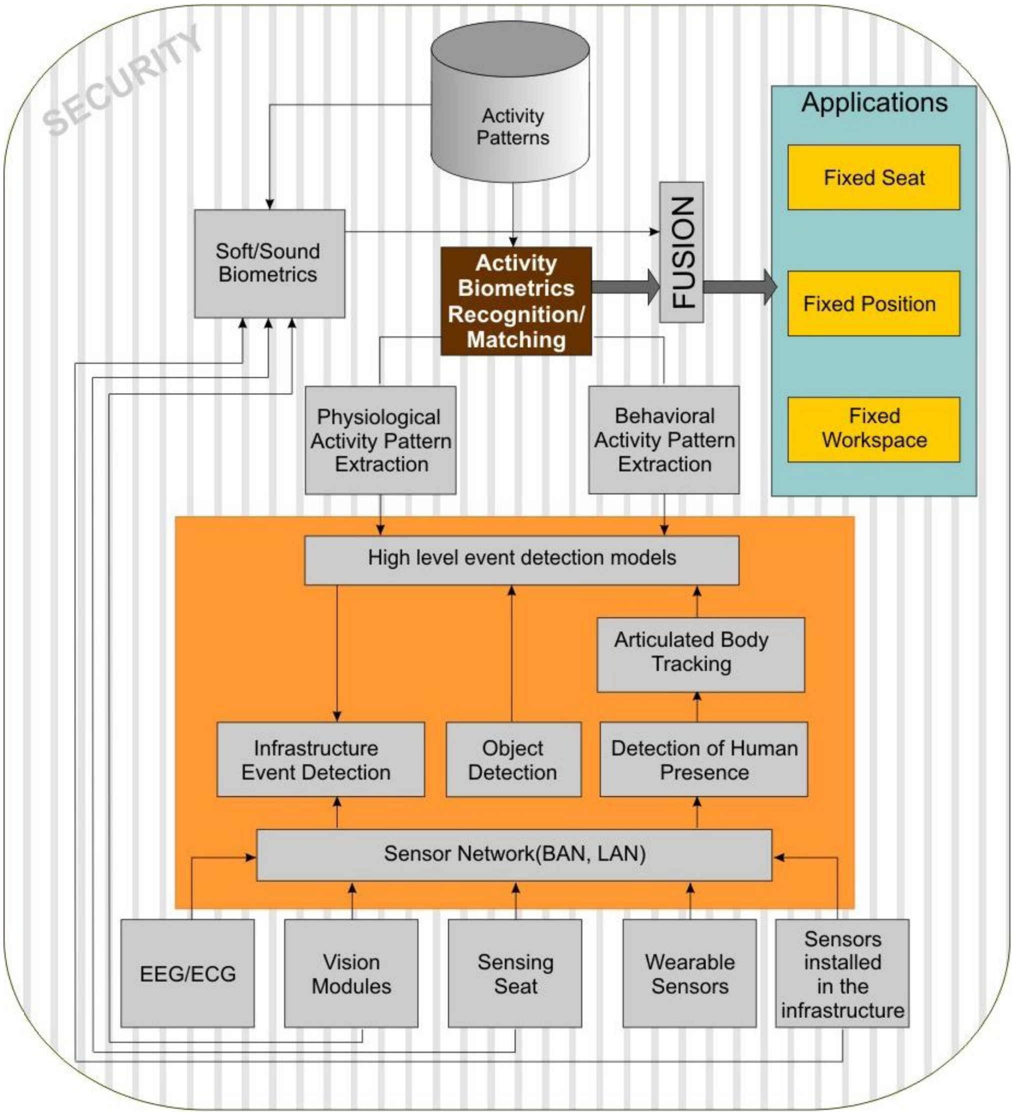

**FIGURE 1.** The ACTIBIO architecture.

The overall architecture of the proposed framework for activity-related biometric authentication integrated into wired and wireless sensor infrastructures is illustrated in Fig. 1. Specifically, the *Sensor Network* is responsible for the collection of data from various heterogeneous sensors, while the *Behavioral Activity Pattern Extraction* module involves the extraction of behavioral activity patterns

(face, gesture, body motion, gait, etc.) by using data from various wireless/wired, wearable/infrastructure-embedded sensors).

Further, the *Physiological Activity Pattern Extraction* module involves the extraction of activity-related and global physiological patterns for authentication (processing EEG, ECG data captured by wireless sensors). The *Activity Biometrics Recognition/Matching* module is the core of the ACTIBIO system, involving multimodal fusion of physiological and behavioral biometric activity-related patterns and the extraction of the activity-related signature that characterizes the individual.

Finally, the module for the detection and tracking of a user is constantly on. The output of this module feeds the *High Level Event Detection Models* that generates a series of events that are then used to extract meaningful activity-related patterns. In this respect, information from the all the modules in the network is received, analyzed, and compared to previously stored activity patterns. When substantial deviation from the stored activity patterns is detected, an alarm is triggered, launching a predefined set of security-related actions.

In addition, issues like the geographical distribution of the system components, data access, data security mechanisms, and compliance with international standards[7] have been taken into account.

### ACTIBIO PILOTS

The effectiveness of the ACTIBIO prototype that integrates all the software and hardware modules is evaluated, and its modularity and adaptation in versatile scenarios is shown in a series of pilots that have been designed as described below. Specifically, three innovative pilots are implemented, targeting the secure "always-on" operation of machines and networked infrastructures controlled by humans (Fig. 2).

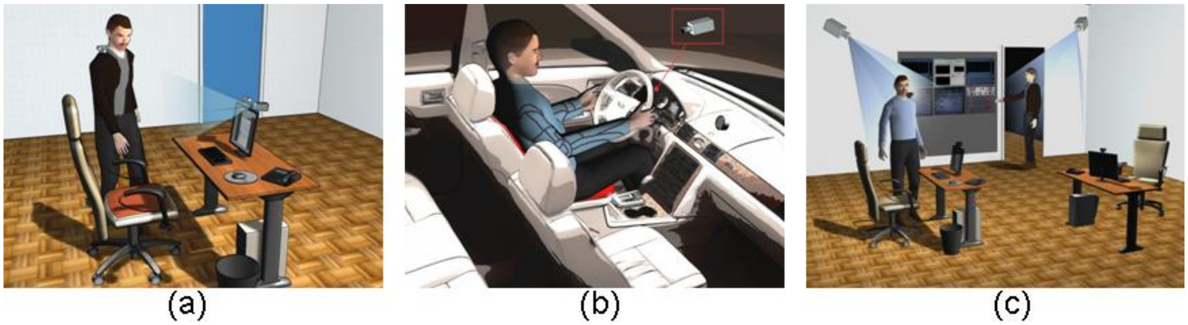

**FIGURE 2**. (a) Fixed-seat pilots, (b) driver pilot, (c) workplace pilot.

These pilots have been planned in such way as to cover the most important aspects of the current project. Moreover, they exhibit its modularity and adaptation in multipurpose scenarios:

1. **A fixed-seat** pilot for enhanced resources protection from unauthorized access and for the evaluation of the system as an emotional state classifier
2. **A driver** pilot representing, in general, the transportation environment
3. **A workplace** pilot for high-security areas and installations protection from unauthorized access and for the evaluation of the system as an emotional state classifier

Table 1 presents the modalities enabled at each of the three ACTIBIO pilots, according to the corresponding project's configurations, so as to highlight the multimodal nature of the system.

**TABLE 1**
**Enable Modalities for Each Pilot**

| ACTIBIO Pilots | Gait | Face Dynamics | Sensing Seat | Physiological (ENOBIO®) | Soft | Activity-Related | Event and Activity Detection |
|---|---|---|---|---|---|---|---|
| Office | × | ✓ | ✓ | ✓ | ✓ | ✓ | ✓ |
| Workplace | ✓ | ✓ | × | (✓) | ✓ | ✓ | ✓ |
| Driver | × | ✓ | ✓ | × | ✓ | × | ✓ |

## THE FIXED WORKPLACE OFFICE PILOT

### The Pilot Protocol

The workplace scenario pilot (Fig. 2c), which demonstrates the unobtrusiveness of the system, is presented in this paper along with some preliminary results for the relevant biometric modalities. The appropriate sensor setup has been used according to the acceptable level of obtrusiveness. In this respect, two scenarios have been considered: (a) the partially obtrusive scenario and (b) the totally unobtrusive scenario, which dictates that the employees do not carry any sensor on them, which in turn means that the physiological profile of the subject was not available.

The system has been installed in a controlled environment in which the individuals were able to move freely in the area of their workspace. The main aim of this pilot is to authenticate the identity of authorized employees that can perform their daily activities, either simple or complex. The subjects have moved in their workspace with no restrictions and several behavioral activity signatures have been automatically extracted. These include body gestures/poses when switching on/off the scene lights, motion analysis of human body shape during walking, dynamic body features extraction during simple activities (e.g., sitting, walking, etc.). Within this environment, the position of the user is detected, while specific actions trigger the system to start recording (e.g., first walk towards the workstation [gait biometrics] and perform simple activities related to the working environment [activity-related biometrics, soft biometrics, activity and event detection]).

The overview of the system that is used for the current scenario is presented in Fig. 3. In the following, each modality is evaluated separately. However, the fusion of several unimodal biometrics is expected to provide enhanced robustness and higher recognition rates. The gait and the activity-related recognition modalities are thoroughly described, while the physiological and face dynamics modalities are described in brief.

### Sensors

The sensors that have been used in the totally unobtrusive version of the workplace scenario are two stereoscopic cameras, one for gait recognition and height estimation, and one for activity-related recognition as well as for soft biometrics of face and face dynamics. Since there were no sensors attached to the subject, the whole process can be characterized as transparent and totally unobtrusive.

On the other hand, the sensors that have been used in the partially obtrusive version of the workplace scenario are the ones above with the addition of minimally obtrusive wearable sensors and the Personal Data Processing Unit (*PDPU*). The wearable sensors are electrodes based on the ENOBIO® technology[8]. These electrodes use nanocarbon substrate to stick to the skin without the need of conductive gel. The ECG signal is then transmitted wirelessly to the *PDPU* for processing and features

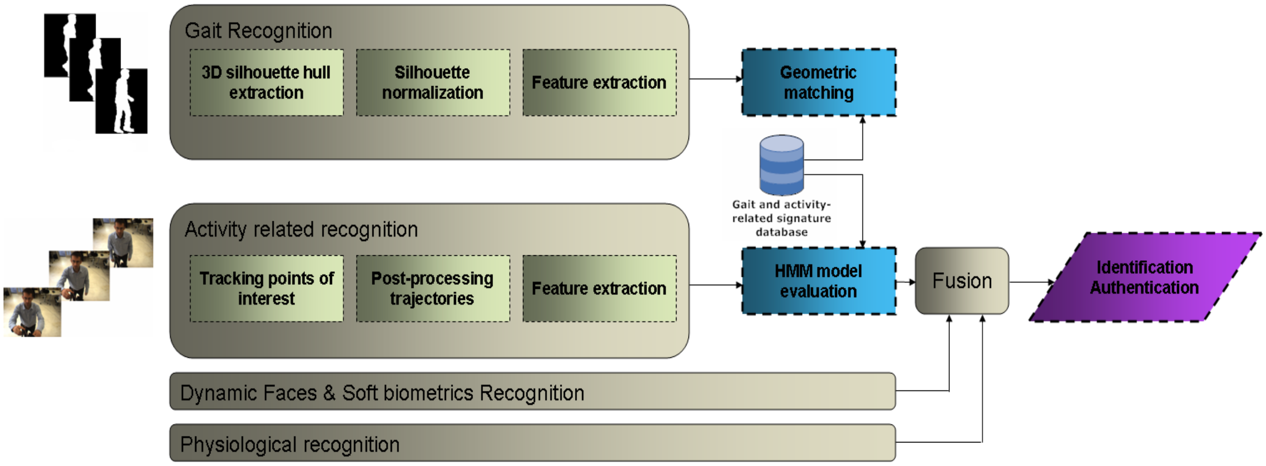

**FIGURE 3.** The full workplace scenario – system overview.

extraction (Fig. 4a). The features are then transmitted to the ACTIBIO system for matching with the corresponding templates. The availability of physiological measurements could potentially be used also for the assessment of the subjects' capacity to perform their task.

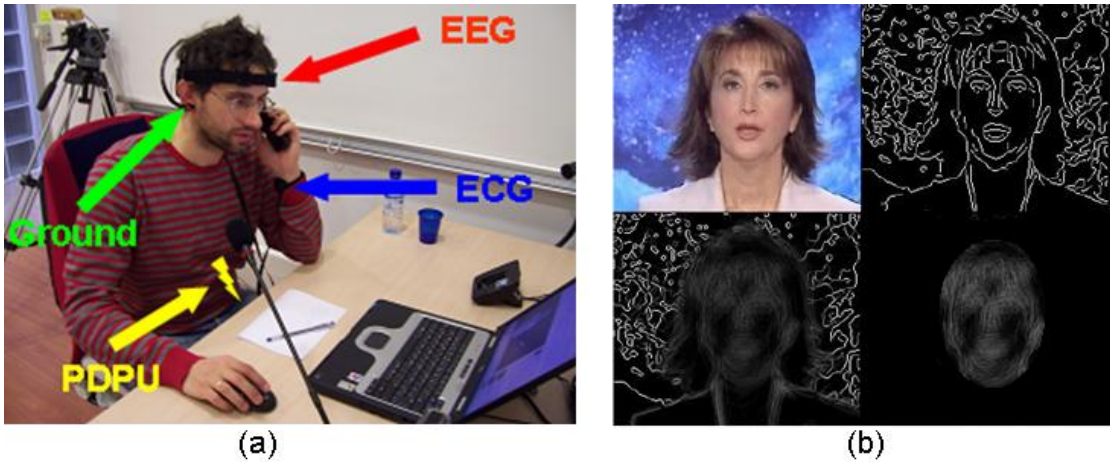

**FIGURE 4**. (a) ENOBIO sensor mounted on a user, (b) extraction of tomofaces over time[9].

### Dynamic Face Recognition Module and Soft Biometrics

Tomofaces[9] is an interesting technique as it uses the edges to compute the facial signature. Tomofaces is more robust to illumination changes than eigenfaces. The reason for this is that edges are less affected by illumination changes than the intensity values of the pixels (i.e., appearance). Moreover, eigenfaces needs heavy preprocessing as it requires the extraction of facial features to perform the face normalization. This is quite a heavy operation in terms of computational power. It is inspired by the research on discrete video tomography[10,11], which applies a temporal X-ray transformation of a video sequence to summarize the facial motion (Fig. 4b).

In addition to the aforementioned technique for extraction of dynamic features from facial images, some soft biometric characteristics can be extracted as well. Specifically, eye color and hair color can easily be derived in conjunction, once the location of the face and the eyes have been processed.

During the feature extraction step, the discriminative information that characterizes the user is preserved, while the rest of the information is discarded. This is achieved by transforming the vectorized X-ray image into the corresponding feature vector. Initially, a linear transformation is applied to reduce the dimension[12] of the X-ray image to a much smaller one. The transformation matrix is computed using the principal component analysis (PCA), which has the ability to represent the distribution of data in the root mean squares sense. The background is removed from the picture, by characterizing as valid only the area indicated by the face detector. Finally, the corresponding feature vector is generated by choosing either the projection in the reduced space or the whitened projection in the reduced space, which rescales the projection to compensate for the overweighting of the low frequencies.

### ACTIBIO Improved Gait Recognition Module

Initially, the walking human binary silhouette is extracted as described in Ioannidis et al.[13]. Let $I_i$ denote the $i^{th}$ binary human silhouette (Fig. 5a). In order to detect stops during a gait sequence, motion estimation through the calculation of a motion history image (MHI)[7] is performed in the silhouette image sequence. Specifically, the motion history template $M_t$ at time instance t is estimated by counting the number of nonzero pixels in the difference image D(I) of two sequential silhouette frames ($I_i$; $I_{i+1}$).

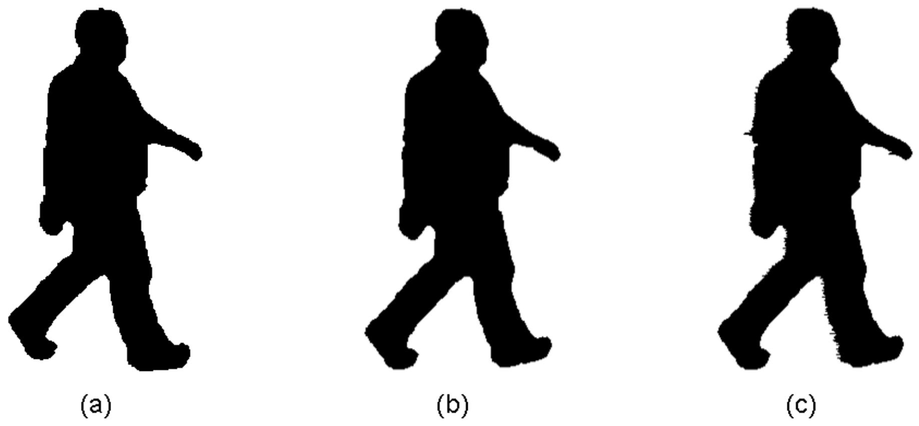

**FIGURE 5**. (a) Originally extracted silhouette, (b) rotated silhouette, (c) rotated silhouette with added emerging points.

The recording phase starts with the detection of silhouette motion in the scene, i.e., when the sum of all nonzero pixels in image $M_t$ exceeds the noise threshold of a nonmotion image. Similarly, a stop in the user's walking is detected when the sum of all nonzero pixels of a corresponding $M_t$ as far as the lower 25% of the silhouette image height, i.e., the part of the legs below the knees[14], is regarded, falls below an experimentally defined threshold. The values for both motion thresholds have been experimentally defined, according to the environmental light conditions.

Once the stop and restart frames are detected, the whole gait cycle that includes the stop frames are removed from the recorded sequence. Thus, a new, cropped set of silhouette sequence is derived. In the following, the gait periods are extracted, as described in Schuckers[3] (Fig. 6a), and the gait cycle indices are estimated accordingly.

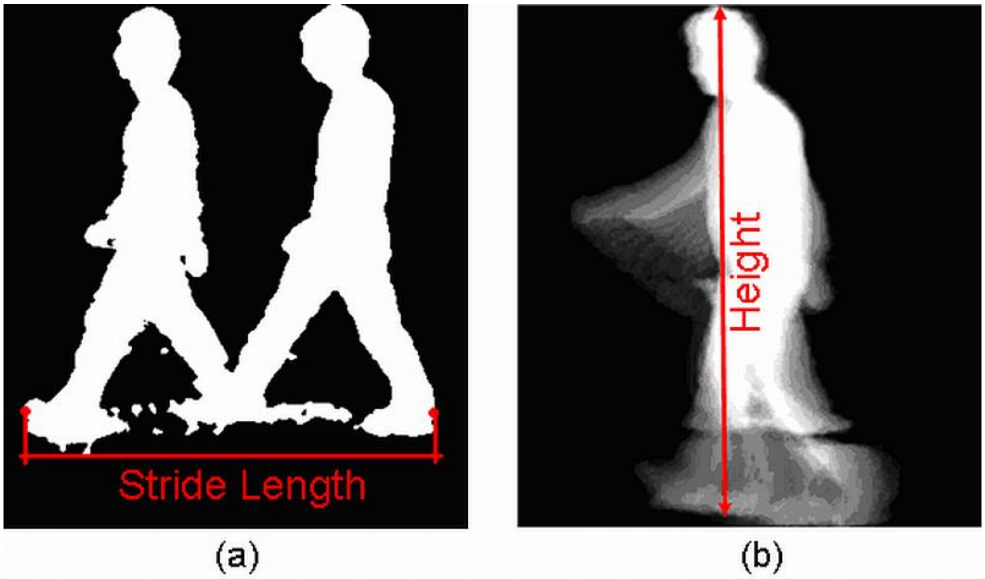

**FIGURE 6.** (a) Estimation of stride length, (b) gait energy image – GEI.

Let the term "gallery" refer to the set of reference sequences, whereas the term "probe sequence" stands for the test sequences to be verified or identified. As reported in the literature, the gait recognition systems achieve high recognition rates when the gallery and the probe sequences demonstrate similar walking angles[15] with respect to the observing camera's local coordinate system. On the contrary, in order to handle cases whereby people walk with arbitrary view angles, different model-based types of angle transformations are applied[16].

However, the accuracy of angle view transformations at model-based approaches relies on small angle variations that are easily affected by slightly noisy images. Thus, a novel feature-based method is introduced in the proposed framework that applies a 3D rotational and reconstruction algorithm on the silhouette itself, encoding shape information about the user's body at the same time, prior to the feature extraction phase. Specifically, range data are used for the compensation of angular variation in the walking direction. The first step is to estimate the relative walking angle. The walking direction with respect to the camera can be estimated in a straightforward manner under the assumption of straight gait within each gait cycle. Given that the highest part of each silhouette image refers to the head of the user, the overall shift of the user in the 3D space within a gait cycle can be estimated just from the first and the last frame of the gait cycle. The walking angle, which is considered constant through each gait cycle, is estimated from the linear processing of the head's 3D locations at the beginning and the end of the cycle.

Thereafter, the silhouettes are rotated so as to register to the frontoparallel view. This is achieved by extracting the 3D coordinates of each silhouette pixel using the disparity data from the stereoscopic camera. This way, a rotated 3D point cloud is generated:

$$P_i^{rotated} = P_i \begin{bmatrix} \cos(\theta) & \sin(\theta) & 0 \\ -\sin(\theta) & \cos(\theta) & 0 \\ 0 & 0 & 0 \end{bmatrix}$$

The new point cloud is now reprojected onto the camera to create a new silhouette (Fig. 5b). The new set of silhouettes $I_{Rot}$ is used to extract the gait features. Despite the notable simplicity of the equation, its direct application in the generation of the virtual view includes some inherent problems related to the fact that reconstructed point clouds could generate nonconsistent surfaces, including holes and nonrealistic edges, when projected onto new virtual views. Therefore, in the proposed framework, a 3D surface is initially formed from the 3D point cloud, so as to generate a consistent surface and silhouette image in the synthesized virtual view (Fig. 5c). The surface is created using only a subset of the joints of the image, so as to reduce the redundancy and size of the triangulated surface to be generated. Then, the silhouette for a particular view is generated by reprojecting it using the Z-buffering principle.

The feature extraction process of the gait sequences is applied on the gait energy images (GEI) and is based on soft biometrics (height), on the radial integration transformation (RIT), and on the Krawtchouk moments (KR) as shown in Fig. 7a and b, respectively. However, instead of applying those transforms on the binary silhouette sequences themselves[13,17], the GEIs (Fig. 6b) are used, which have been proven, on one hand, to achieve remarkable recognition performance and, on the other hand, to speed up the gait recognition[18]. Given the extracted binary gait silhouette images I(i) and the corresponding gait cycles, a GEI is calculated over a gait cycle according to following equation:

$$GEI = \frac{1}{C_L} \sum_{i=cycleStart}^{cycleEnd} I(i)$$

where $C_L$ is the length of the gait cycle and index i refers to the gait cycles extracted in the current gait image sequence. Finally, the RIT and KR transforms are applied on the GEI, after their estimation, in order to construct the gait template for each user.

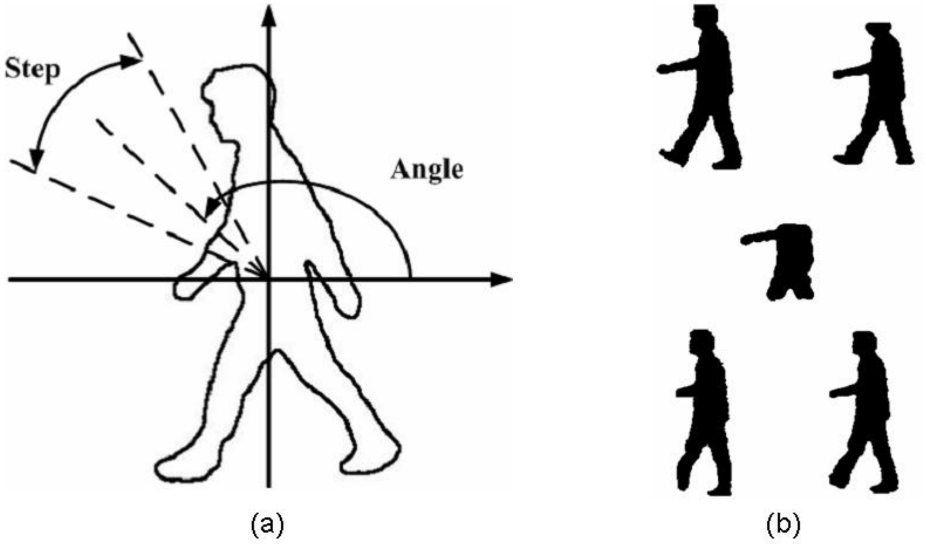

**FIGURE 7.** (a) RIT estimation, (b) KR moments estimation.

In the following, let $G_{GEI}$ and $P_{GEI}$ denote the number of gait gallery and probe cycles, respectively, and T represent a specific feature (RIT or KR). The distance between the probe and the gallery is estimated using the equation below:

$$D_T = \min(Dist_T(1,1),...,Dist_T(i,j))$$

for $i \in [1;GGEI]$ and $j \in [1; PGEI]$, where $Dist_T(i, j)$ is provided by

$$Dist_T(i,j) = \sqrt{| S_T^{Gallery}(i) - S_T^{\text{Pr}obe}(j) |}$$

given that $S^{Gallery}$ and $S^{Probe}$ stand for the value of the corresponding extracted feature (i.e., KR or RIT) for the gallery and the probe collections, respectively.

### ACTIBIO Activity-Related Recognition Module

Activity recognition is performed, again using the concept of MHIs[7]. This temporal template, where the intensity value at each point is a function of the motion properties at the corresponding spatial location in an image sequence, is extracted for each frame, using the last $\tau$ frames. The MHI is then transformed according to the RIT and the circular integration transform (CIT)[19], which are used due to their aptitude to represent meaningful shape characteristics. The location of the head ($x_0$, $y_0$) is detected following the approach in Viola and Jones[20] and is used as the center of integration for both transformations. In particular, the RIT transform of a function *f* is defined as the integral of *f* along a line starting from the center of the image ($x_0$, $y_0$), which forms angle $\theta$ with the horizontal axis (Fig. 8a). In our feature extraction method, the discrete form of the RIT transform is used, which computes the transform in steps of $d\theta$.

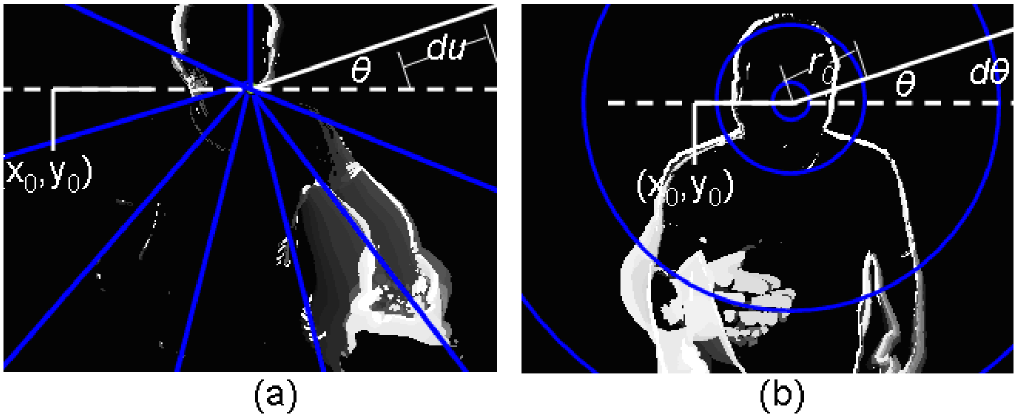

**FIGURE 8**. (a) RIT and (b) CIT for activity detection.

In a similar manner, the CIT is defined as the integral of a function along a circle curve with center ($x_0$, $y_0$) and radius ρ. Similar to the RIT transform, the discrete form of the CIT transform is used, as illustrated in Fig. 8b. The database of supported activities consists of several sets of MHIs transformed according to RIT and CIT methods for each activity. Thus, an incoming transformed signal *x* is compared to a stored one *y* according to two separate classifiers; namely, a Euclidian distance classifier and correlation factor distant curves, as shown in the following equations, respectively:

$$D_E = \sqrt{\| x - y \|^2} \text{ and } corr(x,y) = \rho_{x,y} = \frac{\text{cov}(x,y)}{\sigma_x \sigma_y} = \frac{E((x-\mu_x)(y-\mu_y))}{\sigma_x \sigma_y}$$

The detected event is the one that has the most matches with the prototype MHIs from several subjects, stored in the database, according to a majority voting rule. Accordingly, an activity is considered to be performed within the successive appearance of a starting and an ending event. Moreover, an event is only then detected when the returned scores from both classifiers exceed the experimentally selected thresholds, so as to diminish the false positives.

The aim of the aforementioned activity detection method is the automatic annotation of the full recording. Given that an event has arisen, the annotated frames are segmented from the whole recorded sequence. In the following, they are used as input to the recognition module that is described below.

The user's movements are recorded by a stereo camera and the raw captured images are processed in order to track the users head and hands via the successive application of filtering masks on the captured image[21]. Specifically, when applying a skin-colored mask on all the frames of the extracted activity sequence[22], combined with a motion mask[7], the location of the palms can be provided, while the head can be accurately tracked via a combination of a head-detection algorithm[20] and a mean-shift object tracking algorithm[23]. Thus, the 3D information can be easily derived performing disparity estimation from the input stereoscopic image sequence (Fig. 9).

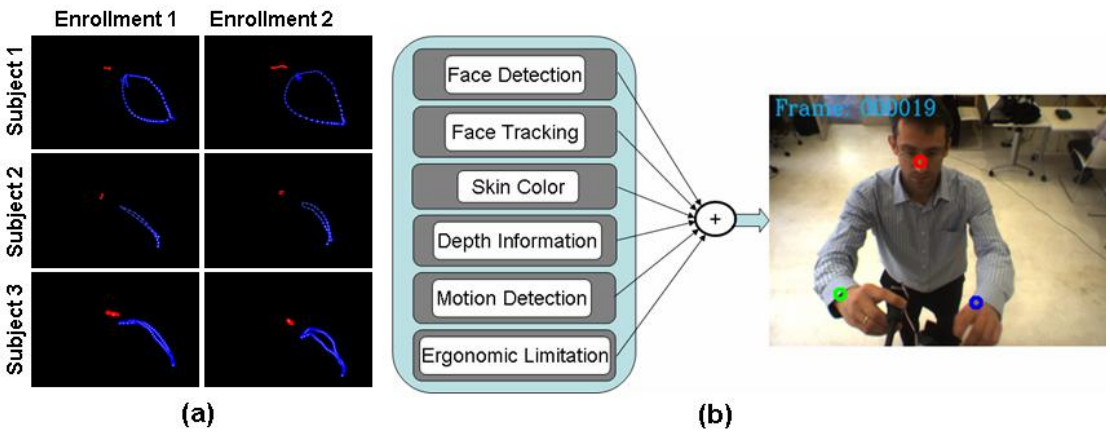

**FIGURE 9**. (a) Extracted trajectories from user's movements, (b) tracking of the user's movement.

Further, a series of postprocessing algorithms[21] applied on the raw tracked points manage to extract smooth motion trajectories that are then used as biometric signatures (Fig. 9). Additionally, in order to provide enhanced invariance with respect to the environmental conditions, the curvature $\kappa(P_l(t))$, the torsion $\tau(P_l(t))$, and their derivatives $\kappa^*(P_l(t))$, $\tau^*(P_l(t))$ for each trajectory are extracted. Specifically, the dependence from the relative position between the camera and the user can be avoided by using view-independent features, as suggested in Wu and Li[24]. In Fig. 10, one can study the noticeable differences of these features between different users with respect to the same movement as far as the trajectory of the right hand is concerned.

A motion trajectory for a certain limb *l* (head or palms) is considered in our work as the 7D N-tuple vector $s_l(t) = (x_l(t); y_l(t); z_l(t); \kappa(P_l(t))); \kappa^*(P_l(t)), \tau(P_l(t)); \tau^*(P_l(t)))$ that corresponds to the x,y,z-axes location of limb center of gravity at each time instance t and the corresponding curvature, torsion, and their derivatives, respectively, of an N-frame sequence. These data of features are then concatenated into a single vector and all vectors produced in a specific activity c form the trajectory matrix Sc. Each repetition of the same activity by a user creates a new matrix. The set of matrices for each user for a specific activity are subsequently used to train a stochastic hidden Markov model (HMM).

Finally, both the training and the identification procedure are implemented by the HMM. Specifically, a five-state, left-to-right, fully connected HMM is trained from several enrollment sessions of the same user. Accordingly, in the verification step, the extracted features from a user are used as input to the stored HMM and the user is classified as client or impostor to the system.

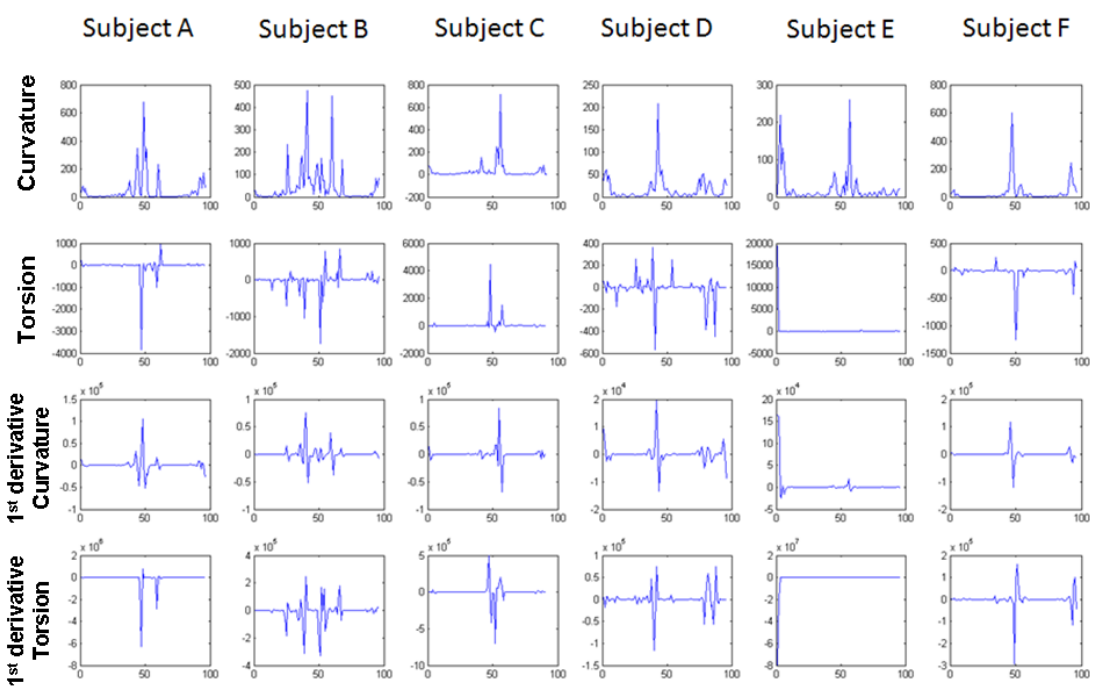

**FIGURE 10.** Trajectories' transformation to view-invariant features.

Last, the same frame sequence is processed so as to extract an estimation for the user's static anthropometric profile that refers to the user's upper-body static skeleton model[21,25] in conjunction to the aforementioned activity-related dynamic features. The full signature for the claimed user ID is restored from the database and the actual extracted features are used to classify the user as a client or an impostor to the system via the HMM classifier, concerning the dynamic motion and an attributed graph matcher (AGM)[26], concerning the static anthropometric information. Finally, a score-level fusion of both classifiers is performed by a support vector machine (SVM) algorithm implemented on a Gaussian kernel and the validity of the final score is then verified by a quality factor based on ergonomic restrictions.

### RESULTS

The proposed framework was evaluated on the publicly available ACTIBIO dataset. This dataset was captured in an AmI indoor environment and its recordings include 29 subjects, performing a series of office/workplace activities and walking in both straight and arbitrary paths for eight repetitions each. The testing conditions for the reported results for the rest of the modalities are described below.

#### Face Biometrics

The model estimation for the tomofaces is similar to that of the eigenfaces technique[12]. Each person model is characterized by points in the feature space that summarize the distribution of the feature vectors of each person. In other words, the cluster center of the feature vectors is computed by taking the average (centroid) or the median feature vector.

The authentication decision is made using the concept of the nearest-neighbor classifier. The unknown feature vector is compared to all the client models and selects the best match using one of the following distance metrics: city-block distance, Euclidean distance, or cosine distance. The EER (equal error rate) score derived from the current method is close to 8% in the aforementioned database.

### Physiological Biometrics

Regarding EEG and ECG modules, the main difference with the HUMABIO (Human Monitoring and Authentication using Biodynamic Indicators and Behavioral Analysis) approach[27] is that, in this case, an improved recording protocol is used in conjunction with wavelet transforms and linear discriminant analysis (LDA) classifying techniques. Specifically, after applying the artifact corrector, it can be noted that the results are significantly encouraging, lying at around 22%

With EEG, the authentication scores are ~18% of EER after the artifact correction. These EERs are obtained by averaging the EER over the three office takes. Although more takes would be better to have a meaningful performance, it should be noted that for each take, 29 subjects have been used. In the computation of the EER, each subject claims to be all the subjects of the database (one legal transaction and 28 impostor transactions). Therefore, the above averages are a meaningful way to compute the performance of our systems.

In the same concept, the results of the ECG biometric module, after having applied the artifact detector module, have been found to be ~30%. Both EEG and ECG have been tested while the subject was walking. The recognition potential from the office recording (the user remained seated) is expected to be much higher, since the movement artifacts are much stronger when the subject is walking than just sitting at his desk. Fusing these results with the other modalities within ACTIBIO would definitively increase the performance.

### Gait Biometrics

The insertion of the GEI concept in conjunction with the use of the proposed gait recognition modality (i.e., stop detection and silhouette rotation), as well as the improved RIT and KR algorithms classifiers, provide significant improvements in the gait recognition module in comparison to the HUMABIO approach[28].

Specifically, one can notice that the significant influence of the rotation compensation algorithm can be demonstrated compared to the method proposed in Ioannidis et al.[13]. In particular, the recognition rates have increased by a mean ratio of 20% (peek ratio improvement 35%) in the RIT classifier case, and by a mean ratio of 10% (peek ratio improvement 23%) in the KR classifier case. Furthermore, with the additional use of the stop detection algorithm, the recognition rates increase even more, reaching a ratio value of 15.9 and 16.5% in both the RIT and KR classifier cases, respectively.

In the same respect, the current enhancements exhibit high authentication scores, given the EER results in Table 2.

**TABLE 2**
**Gait Authentication Rates**

| | Gait (RIT) | Gait (KR) | Fusion |
|---|---|---|---|
| EER | 15.9% | 16.5% | 11.7% |

### Activity Related Biometrics

Given the high degrees of unobtrusiveness of the method, significantly high authentication rates can be noted in the recognition performance when augmenting the existing algorithm with the static anthropometric information. The proposed framework has been evaluated in the context of three verification scenarios. Specifically, the potential of user's authentication has been tested, based on the activity-related signature during the same activity when the user interacted with the workplace in front of him/her. The EER in this scenario was found to be 13%.

As expected, the authentication performance of the system improves further when combining both modules. Namely, with the contribution of both the gait module and the activity-related module, an EER of ~8% has been calculated. Specifically, for the score-level fusion, the SVM classifier has been used based on a Gaussian kernel with a width value of 0.01. Further, the trade-off factor between training error and margin was set at 100,000, while all input score data have undergone the "min-max" normalization.

### Activity Detection

The performance of the proposed activity detection framework exhibited high accuracy, as described in the matrix of Table 3. The performed experiment forced a simultaneous search for the detection of four different activities. Namely, the activities of a "phone conversation", an "interaction with an office panel", the "talking into a microphone", and the "insertion of a card into a slot" have been simultaneously looked for at each frame.

**TABLE 3**
**Event and Activity Detection Rates**

| Event | Phone Conversation | Panel Interaction | Talking into Microphone | Card Insertion |
|---|---|---|---|---|
| Phone conversation | 93.1% | 0% | 0% | 6.9% |
| Panel interaction | 0% | 89.7% | 10.3% | 0% |
| Talking into microphone | 0% | 10.3% | 86.2% | 3.44% |
| Card Insertion | 0% | 3.44% | 3.44% | 93.1% |

Activities that gather a lot of energy in the same areas of the frame are most likely to be mismatched, i.e., the microphone and panel are on the same side of the user and both require the user to lean towards them. On the other hand, activities performed on distinctive areas of the image, i.e., the users picks the phone with the left hand and speaks into the microphone on his right side, are more likely to be correctly detected.

### DISCUSSION

Behavioral biometrics are very valuable for many realistic application scenarios that require identification or authentication of an individual. Behavioral biometrics are often employed because they can be easily collected unobtrusively and are particularly useful in situations that do not provide an opportunity for collection of stronger, more reliable, biometric data. Moreover, they demonstrate great adaptability to current security installations since just a couple of cameras are required.

In this respect, the ACTIBIO approach offers increased safety and enhanced security solutions in AmI infrastructures. Some potential applications that can be empowered by the ACTIBIO project are aiming at health monitoring; environmental control of secure installations or databases; and monitoring of entire houses, including all major appliances and the access device status, and controlling them from anywhere in the world. In particular, ACTIBIO can be adjusted in order to enhance alarm detection and security against trespassing, as well as to ensure that only authorized persons have access to health information data.

Regarding the health-oriented applications, there is sufficient evidence that medical conditions can be detected by behavioral biometrics. Thus, behavioral biometrics, such as gait or other activity-related biometrics, can contribute in the examination of detected medical conditions/disabilities (i.e., orthopedic problems). Furthermore, behavioral biometrics, using body dynamics, could potentially detect (a) psychiatric conditions, such as dissociative disorders, acute anxiety, panic, and major depression; (b) neurological conditions, such as movement disorders; (c) musculoskeletal and articular disorders, such as foot and ankle disorders (gait), joint disorders (activity related); and (d) all those conditions that can generate symptoms similar to those of previous disorders.

Additionally, health and social care institutions/organizations could avoid congestion of patients with automatic remote monitoring of the condition of the latter. Given that there are many people with severe disabilities who face difficulties in gaining access to appropriate services, housing, etc., they are usually constrained either to rely on the assistance of their relatives or to bear a very limited standard of living. Consequently, it is possible to moderate the role of the above institutions/organizations and, conclusively, to reduce the health costs that the handicapped people and their families bear. Accordingly, health care institutions and organizations will highly benefit from the introduction of novel tools that minimize the time, cost, as well as the need for personnel.

### CONCLUSION

In this paper, a novel biometric authentication system, developed within the framework of the ACTIBIO project, has been presented. It has used novel behavioral traits in combination with state-of-the-art algorithms, aiming primarily at the user's convenience and unobtrusiveness. Novel biometric modalities have been studied and used in order to overcome several shortcomings of the current biometrics solutions, mainly, the strict protocols required to be followed by the subjects. ACTIBIO, among other innovations, will support authentication of the individuals in a continuous way and will also allow the monitoring of the physiological parameters to ensure the normal state of critical process operators. Its three pilots are designed in such a way as to demonstrate the versatility and extensive modularity of the system, and to provide performance evaluation in realistic application scenarios.

### ACKNOWLEDGMENTS

This work was supported by the EU funded ACTIBIO ICT STREP (FP7-215372).

## References

1. Qazi, F.A. (2004) A survey of biometric authentication systems. *Security Manag.* 61–67.
2. Xiao, Q. (2005) Security issues in biometric authentication. *Inf. Assur. Workshop, IAW* 8–13.
3. Schuckers, S.A.C. (2002) Spoofing and Anti-Spoofing Measures. *Inf. Security Tech. Rep.* **7(4),** 56–62.
4. Fairhurst, M.C., Deravi, F., Mavity, N., George, J., and Sirlantzis, K. (2003) Intelligent management of multimodal biometric transactions. *Lect. Notes Comput. Sci.* **2774,** 1254–1260.
5. Snelick, R., Uludag, U., Mink, A., Indovina, M., and Jain, A. (2005) Large-scale evaluation of multimodal biometric authentication using state-of-the-art systems. *IEEE Trans. Pattern Anal. Mach. Intell.* **27(3),** 450–455.
6. Ferro, M., Pioggia, G., Tognetti, A., Carbonaro, N., and De Rossi. D. (2009) A sensing seat for human authentication. *IEEE Trans. Inf. Forensics Security* **4(3),** 451–459. http://www.actibio.eu:8080/actibio/results/publications.html

7. Bobick, A. and Davis, J. (2001) The recognition of human movement using temporal templates. *IEEE Trans. Pattern Anal. Mach. Intell.* **23(3),** 257–267.
8. Ruffini, G., Dunne, S., Farrés, E., et al. (2006) ENOBIO—first tests of a dry electrophysiology electrode using carbon nanotubes. In *Proceedings of the 28th Annual International Conference of the IEEE Engineering in Medicine and Biology Society (EMBS '06), New York, August*. IEEE. pp. 1826–1829.
9. Ouaret, M., Dantcheva, A., Min, R., Daniel, L., and Dugelay, J.L. (2010) BIOFACE, a biometric face demonstrator. In Proceedings of the International Conference on Multimedia, *October 25–29, Florence, Italy.* ACM. pp. 1613–1616.
10. Akutsu, A. and Tonomura, Y. (1994) Video tomography: an efficient method for camerawork extraction and motion analysis. In *Proceedings of the Second ACM International Conference on Multimedia '94, October 15–20, San Francisco.* ACM Press. pp. 349–356.
11. Joly, P. and Hae-Kwang, K. (1996) Efficient automatic analysis of camera work and microsegmentation of video using spatio-temporal images. *Signal Process. Image Commun.* **8(4),** 295–307.
12. Turk, M.A. and Pentland, A.P. (1991) Face recognition using eigenfaces. *Proceedings of the 1991 IEEE Computer Society Conference on Computer Vision and Pattern Recognition (CVPR), June 3–6, Maui, HI.* IEEE. pp. 586–591.
13. Ioannidis, D., Tzovaras, D., Damousis, I.G., Argyropoulos, S., and Moustakas, K. (2007) Gait recognition using compact feature extraction transforms and depth Information. *IEEE Trans. Inf. Forensics Security* **2(3),** 623–630.
14. Goffredo, M., Bouchrika, I., Carter, J.N., and Nixon, M.S. (2010) Self-calibrating view-invariant gait biometrics. *IEEE Trans. Syst. Man Cybern. B Cybern*. **40(4),** 997–1008.
15. Sarkar, S., Phillips, P.J., Liu, Z., Robledo-Vega, I., Grother, P., and Bowyer, K.W. (2005) The human ID gait challenge problem: data sets, performance, and analysis. *IEEE Trans. Pattern Anal. Mach. Intell.* **27(2),** 162–177.
16. Goffredo, M., Bouchrika, I., Carter, J.N., and Nixon, M.S. (2009) Performance analysis for automated gait extraction and recognition in multi-camera surveillance. *Multimedia Tools Appl.* **50(1),** 75–94.
17. Ioannidis, D., Tzovaras, D., and Moustakas, K. (2007) Gait identification using the 3D Protrusion Transform. In *IEEE International Conference on Image Processing, ICIP 2007, September 16 to October 19, San Antonio, TX.* Vol. 1. pp. 349–352,
18. Yu, C., Cheng, H., Cheng, C., and Fan, K.-C. (2010) Efficient human action and gait analysis using multiresolution motion energy histogram. *EURASIP J. Adv. Signal Process.* 13 p.
19. Simitopoulos, D., Koutsonanos, D.E., and Strintzis, M.G. (2003) Robust image watermarking based on generalized Radon transformations. *IEEE Trans. Circuits Syst. Video Technol.* **13(8),** 732–745.
20. Viola, P. and Jones, M. (2001) Rapid object detection using a boosted cascade of simple features. In *Proceedings of the 2001 IEEE Computer Society Conference on Computer Vision and Pattern Recognition (CVPR), December 8–14, Kauai, HI.* Vol. 1. IEEE. pp. 511–518.
21. Drosou, A., Moustakas, K., and Tzovaras, D. (2010) Event-based unobtrusive authentication using multi-view image sequences. In *Proceedings of the First ACM International Workshop on Analysis and Retrieval of Tracked Events and Motion in Imagery Streams (ARTEMIS), October 25–29, Florence, Italy.* ACM. 94 p.
22. Gomez, G. and Morales, E.F. (2002) Automatic feature construction and a simple rule induction algorithm for skin detection. In *Proceedings of the ICML Workshop on Machine Learning in Computer Vision (MLCV), July 9, Sydney, Australia.* pp. 31–38.
23. Comaniciu, D., Ramesh, D.C.V., and Meer, P. (2000) Real-time tracking of non-rigid objects using mean shift. In *Proceedings of the 2000 IEEE Computer Society Conference on Computer Vision and Pattern Recognition* (CVPR*), June 13–15, Hilton Head, SC.* Vol. 2. IEEE. pp. 142–149.
24. Wu, S. and Li, Y.F. (2009) Flexible signature descriptions for adaptive motion trajectory representation, perception and recognition. *Pattern Recognition* **42,** 194–214.
25. Alcoverro, M., Casas, J.R., and Pardas, M. (2010) Skeleton and shape adjustment and tracking in multicamera environments. In *Articulated Motion and Deformable Objects. 6th International Conference, AMDO 2010. Port d'Andratx, Mallorca, Spain, July 2010, Proceedings.* Perales, F.J. and Fisher, R.B., Eds. Springer. p. 88.
26. Wyk, B.V. and Wyk, M.V. (2003) Kronecker product graph matching. *Pattern Recognition* **36(9),** 2019–2030.
27. Damousis, I.G., Tzovaras, D., and Bekiaris, E. (2008) Unobtrusive multimodal biometric authentication: the HUMABIO project concept. *EURASIP J. Adv. Signal Process.* Article No. 110.
28. HUMABIO ICT STREP (2006) www.humabio-eu.org/.
